# Characterization of the complete chloroplast genome of *Carya laciniosa* (F.Michx.) G.Don (Juglandaceae)

**DOI:** 10.1080/23802359.2021.1906178

**Published:** 2021-04-09

**Authors:** Min Zhai, Xiao-Dong Jia, Ji-Yu Zhang, Zhong-Ren Guo, Ji-Ping Xuan

**Affiliations:** a Institute of Botany, Jiangsu Province and Chinese Academy of Sciences, Nanjing, PR China; b The Jiangsu Provincial Platform for Conservation and Utilization of Agricultural Germplasm, Nanjing, PR China; c Jiangsu Key Laboratory for the Research and Utilization of Plant Resources, Nanjing, PR China

**Keywords:** *Carya laciniosa*, chloroplast genome, phylogenetic analysis

## Abstract

*Carya laciniosa* (F.Michx.) G.Don is a slow-growing valuable tree in the family Juglandaceae. In this research, we first reported the chloroplast genome sequence of *C. laciniosa*. The chloroplast genome was a circular form with 160,832 bp in size, comprising four subregions: a large single-copy (LSC) region of 90,065 bp, a small single-copy (SSC) region of 18,791 bp, and two copies of inverted repeats (IRs; IRa and IRb) of 25,988 bp each. A total of 132 genes were annotated in the chloroplast genome of *C. laciniosa*, including 87 protein-coding genes, 37 tRNA genes, and 8 rRNA genes. The overall GC content of the whole genome was 36.14%. Phylogenetic analysis suggested that *C. laciniosa* was closely related to *C. ovata*.

*Carya laciniosa* is a slow-growing long-lived tree in Juglandaceae family with high value (Grauke et al, 2016). Its wood is hard, strong, and flexible, which could be used for furniture, tool handles, and sporting goods (Sargent 1918). Additionally, its nuts are sweet and edible, and have the largest nut size among the hickories (Sargent 1918). More importantly, *C. laciniosa* could be hybridized with *C. illinoinensis* and *C. cathayensis* (Juglandaceae species with high economic values) (Thompson and Grauke 1991), making it an important genetic material for cross-breeding. Nowadays, the genomic data regarding *C. laciniosa* still needs further investigation. Chloroplast genome is a circle molecular that could be divided into four subregions: a large single-copy (LSC) region, a small single-copy (SSC) region, and two inverted repeat (IR) regions (Du et al. 2017). Furthermore, a typical angiosperm chloroplast genome has a size ranging from 115 to 165 kb (Sun et al. 2020). Owing to its a relatively simple structure and small size, chloroplast genome data has been used extensively for phylogenetic analysis and species identification (Hong et al. 2017; Li et al. 2018). In this study, we assembled the complete chloroplast genome of *C. laciniosa*, and analyzed its phylogenetic relationship.

Fresh Leaves of *C. laciniosa* were sampled from the experimental farm of Nanjing Botanical Garden (Nanjing, China 118°51′12.73″E, 32°2′11.28″N), and were deposited at Institute of Botany, Jiangsu Province and Chinese academy of sciences (Voucher specimen No. Zhai20200813). Whole genomic DNA was extracted using Tiangen Plant Genomic DNA Kit (Tiangen Biotech Co., Beijing, China). Paired-end libraries with insert sizes of 300 bp were constructed using an Illumina Hiseq Library Kit (Illumina, San Diego, CA) following the manufacturers’ procedure. The genome sequencing was done on an Illumina Hiseq 4000 platform (Illumina, San Diego, CA). Following sequencing, the Hiseq4000 generated about 6 GB raw data with 71.4 million reads (NCBI SRA accession number SRR13222018). Raw reads were then filtered by Trimmomatic version 0.36 software (Bolger et al. 2014) with default parameters to obtain clean reads. NOVOPlasty software (Dierckxsens et al. 2017) was used to assemble the chloroplast genome based on clean data. Chloroplast genome annotation was performed by GeSeq (Tillich et al. 2017) with *C. ovata* (MT701613; Xu et al. 2020) as a reference, and manually checked by Geneious Prime (Kearse et al. 2012). The annotated chloroplast genome has been deposited in GenBank under the accession number MW186783.

The chloroplast genome exhibited a quadripartite structure with lengths of 160,832 bp, in which a LSC region (90,065 bp) and a SSC region (18,791 bp) were separated by a pair of IRs (IRa and IRb) region (25,988 bp, each). The overall GC content of *C. laciniosa* chloroplast genome was 36.14%; IRs regions had a relatively high GC content (42.59%), followed by LSC (33.73%) and SSC (29.89%) regions. The chloroplast genome encoded a total of 132 genes, including 87 protein-coding genes, 37 tRNAs, and 8 rRNAs. There were 19 genes duplicated in the IR region, including 8 protein-coding genes, and 11 other genes. Additionally, 11 intron-containing genes were detected with 9 genes containing one intron and 2 genes (*ycf3* and *clpP*) containing two introns.

The chloroplast genomes of 17 Juglandaceae species (including *C. laciniosa* in this study) were selected for phylogenetic analysis. Multiple sequence alignment was performed using MAFFT software (Katoh and Standley 2013). Phylogenetic analysis was conducted by maximum likelihood (ML) methods with a bootstrap of 1000 repetitions. ML phylogenetic tree was implemented with IQ-TREE (Nguyen et al. 2015) (Figure 1). Based on morphological characteristics, there are three sections in the genus *Carya*, including sect. *Apocarya*, sect. *Carya*, and sect. *Sinocarya* (Manos and Stone 2001). *C. laciniosa* and *C. ovata* are the representative of sect. *Carya*. Our phylogenetic analysis suggested that *C. laciniosa* was closely related to *C. ovata*, which supported the current morphological classification.

**Figure 1.**
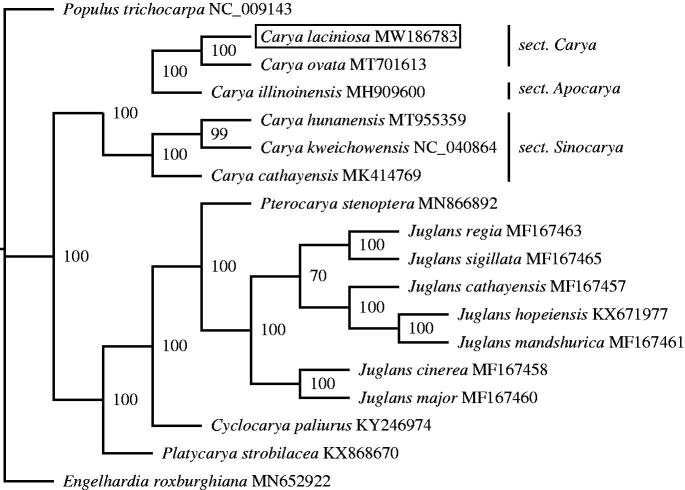
Phylogenetic tree construction using maximum likelihood (ML) based on the complete chloroplast genomes of 17 Juglandacea species. NumberS at the tree nodes represent bootstrap support values based on 1000 replicates.

## Data Availability

The genome sequence data that support the findings of this study are openly available in GenBank of NCBI at [https://www.ncbi.nlm.nih.gov] (https://www.ncbi.nlm.nih.gov/) under the accession no. MW186783. The associated BioProject, SRA, and Bio-Sample numbers are PRJNA683718, SRR13222018, and SAMN17035824 respectively.
